# Outpatients’ perception of their preoperative information regarding their health literacy skills and their preoperative anxiety level: Protocol for a prospective multicenter cross-sectional study

**DOI:** 10.1097/MD.0000000000026018

**Published:** 2021-05-21

**Authors:** Chandler-Jeanville Stephanie, Ahouah Mathieu, Margat Aurore, Monique Rothan-Tondeur Monique

**Affiliations:** a Sorbonne Paris Nord University, Chaire Recherche Sciences Infirmières, LEPS; b Assistance Publique Hôpitaux de Paris, Avicenne Hospital, Hôpitaux Universitaires Paris Seine-Saint-Denis, Anesthesia Department, Bobigny, France.

**Keywords:** ambulatory surgery, health literacy, patient experience, preoperative anxiety, preoperative instructions

## Abstract

Despite the benefits related to ambulatory surgery such as cost reduction due to lack of accommodation and patient satisfaction due to early home return, it may not lead to these expected benefits. Indeed, this kind of practice can increase responsibility for the person being treated and his or her relatives. It is therefore essential to inform them as well as possible to obtain their adherence to the proposed care protocol. Nevertheless, patients’ failures to comply with preoperative instructions or the non-attendance of the patient may result in late cancellation of the scheduled surgery. In order to reduce this kind of dysfunction, the Assistance Publique-Hôpitaux de Paris (APHP) uses a reminder system by Short Message Service (SMS).

This study is a descriptive cross-sectional multicenter study that focuses on outpatients’ lived experiences of their preoperative preparation and information. It aims to collect patients’ perceptions of their ability to follow preoperative instructions received by SMS the day before an operation performed for ambulatory surgery, according to their level of health literacy (HL) and preoperative anxiety. Indeed, poor communication between patients and doctors can contribute to preoperative anxiety, while low health literacy (LHL) can lead to poor understanding of preoperative preparation instructions. Therefore, it seems important to take these 2 criteria into account in this study. This research is designed to interview outpatients undergoing ambulatory surgery in the establishments of APHP. A self-questionnaire will be used for this purpose. The choice of this institution is justified by its decision to use in all care units the reminder of preoperative instructions by SMS.

The main outcome is the perception of outpatients with LHL skills regarding preoperative information provided by doctors.

French ethics review committee (Comité d’Ethique de la Recherche) of the University of Paris has approved the study protocol (IRB 00012020-14). Results from this study will be disseminated through oral communications and a scientific article in an international peer-reviewed journal.

This protocol is registered on researchregistry.com (researchregistry5834). This version number is 1.1 Protocol dated July 22, 2020.

## 1 Introduction

Each year, more than 310 millions operations are performed worldwide.^[1]^ Amongst them, outpatient surgical procedures have gained interest and their volume has been increasing over the past 40 years, especially in most of high-income countries where it exceeds the inpatient procedures. Indeed, in countries like United States of America (USA) and United Kingdom (UK), pioneers in its development,^[2]^ day surgery rate has come from 34% to 61% between 1985 and 1994 in USA and from 15% to 70% in the UK from 1989 to 2003. However, in other countries, like France, the day surgery rate has increased slower, and it is only since 2013^[3]^ that more than half of the surgical acts (50.4%) are performed this way according to the Technical Agency on Hospitalization Information (Agence Technique de l’Information sur l‘Hospitalisation). Nevertheless, despite this growth disparities, various national health agencies^[4]^ promote and support day surgery development with the target that about 70% of elective surgery should be performed as day surgery procedures.

But the shift from inpatients to ambulatory surgery represents a huge challenge for healthcare structure, as it implies patients undergoing surgery do not require overnight stay and are discharged the same day of surgery.^[5]^ Therefore, the efficiency of day surgery requires the patient active participation at every step of his pathway, which implies his responsibility throughout his care, which begins and continues outside the hospitals. Actually, in this particular context, outpatients must be partners of healthcare providers through shared decision making (SDM), because a lack of engagement in their care can harm their safety and thus, day surgery performance.^[6]^ Furthermore, failures can occur in the preoperative period due to nonrespect of preoperative instructions, which can lead to reschedules or late cancellations on the day of surgery.

To avoid these adverse events, more and more hospitals, like the ones of the Assistance Publique–Hôpitaux de Paris group, use Short Service (SMS) reminders. This way, they provide outpatients with standard preoperative instructions.^[7,8]^ However, different studies have shown that tailored preoperative information is essential for helping the outpatient to cope with day surgery process, by becoming engaged in his care.^[9]^

But, standard preoperative information does not fit all outpatients. Indeed, for an effective delivery, healthcare providers should be aware of factors, which can lead to perioperative vulnerability as low health literacy (LHL) skills and a high level of preoperative anxiety.

## 2 Background

### 2.1 Patients’ perioperative vulnerability: an issue with adverse effects on surgical performance

Having a surgery often goes with a powerlessness feeling for the patient, who perceive a breakthrough of his autonomy surrounding by the “hostile” environment^[10]^ of the operating room.

This can lead to perioperative vulnerability, which can have 3 dimensions: social, physical and psychological.^[11]^ Physical vulnerability comes with the inability for the patient to handle a worsening of their health status, in particular when their physiological state is already altered like with ageing.^[11]^

Social vulnerability refers to demographic, economic and cultural factors which can contribute to a low level of health literacy.^[12]^ LHL skills are associated with poor outcomes in health, as in surgery.^[13]^ The WHO defines health literacy (HL) as “the cognitive and social skills which determine the motivation and ability of individuals to gain access to, understand and use information in ways which promote and maintain good health”.^[14]^ Actually, people with LHL skills are less likely to follow preoperative medical instructions because they are not able to fully understand them,^[13]^ which can lead them to be less adherent to the proposed care pathway.^[12]^ Moreover, patients with LHL skills can be more passive during SDM process.^[15]^

Psychological vulnerability relates to the negative emotions (anxiety, fear), which can disrupt patient's self-esteem, especially when he is confronted to an unknown place like the operating room.^[11]^ Sixty percent to 80% of surgical patients can suffer from preoperative anxiety.^[16]^ High level of preoperative anxiety can harm the anesthetic^[17–19]^ and surgical care,^[20,21]^ and can thereby extend the outpatients’ length of stay.

If healthcare providers cannot reverse ageing, they can act on social and psychological vulnerability by tailoring preoperative information.

### 2.2 Day surgery: a challenge for satisfying outpatients’ needs of information

Psychological preoperative preparation is one of the ways healthcare providers, especially nurses, can use to help patients to cope with the powerlessness induced by the surgical pathway. Indeed, preoperative psychological preparation is “a range of strategies designed to influence how a person feels, thinks or acts.”^[22]^ Thereby, psychological preoperative preparation aims to ease patients’ engagement and to reduce preoperative anxiety without medications.

Healthcare providers can use this intervention to deliver outpatients the right information (in terms of content and of amount) according to their psychological profile.^[23]^

Moreover, through day surgery pathway, preoperative information is essential because it enables the patient to be fully aware of every step of his pathway and to ease his engagement in his care. Patient engagement refers to “the desire and capability [for the patient] to actively choose to participate in care in a way uniquely appropriate to the individual, in cooperation with a healthcare provider or institution, for the purposes of maximizing outcomes or improving experiences of care.”^[24]^ However, standard information does not fit every patient, thus, healthcare providers should be aware of outpatients’ specific needs when delivering preoperative information.^[25]^ Indeed, since 1994, the WHO has encouraged patients’ rights with the Declaration on promotion of Patients’ rights in Europe. It results in a statement,^[26]^ which promoted their rights “to be fully informed about their health status,” and about the features of their care (risks and benefits, different alternatives to the proposed treatment).

This right to be informed is the cornerstone of SDM, which implies that the healthcare providers are aware of the constraints that may influence preoperative information delivery. Therefore, through day surgery process, healthcare providers often use verbal communication for providing preoperative information. However, limitations can occur in this context, because healthcare providers, especially nurses, are less available for providing preoperative information to outpatients.^[27]^

Actually, through surgical pathway, a patient can meet up to 27 different healthcare providers,^[28]^ which can lead to inadequate or insufficient information because the preoperative information's contents and quality varied.^[29]^ Furthermore, nurses recognize the provided information do not always meet outpatients’ expectations,^[27]^ which can contribute to raise preoperative anxiety level thus we know that 82% of surgical patients wish to have more knowledge about their care.^[30]^ Indeed, different studies have shown that lack of information is one of the main complaints of day surgery patients.^[15,31]^ Therefore, healthcare providers use other supports like booklets, movies, websites, to complete preoperative information.^[29]^ However, people with LHL may not understand them,^[32,33]^ which can lead to delays or cancels the elective surgery.

### 2.3 The ineffective management of patient-related cancellations in day surgery

Surgical care efficiency implies a specific organization, especially in day surgery. However, late cancellations or day of surgery (DOS) cancellations can occur, and their rate is an indicator to assess perioperative care quality.^[34]^ Although, they are lower in day surgery^[35,36]^ compared to inpatients surgical procedures, DOS cancellations in day surgery are partly patient-related.

Indeed, patient no show is the main reason of DOS cancellations, followed by inadequate preoperative preparation and health status change.^[35]^ The explanations of patients no show are often linked with psychological factors (like doubts and fears about the surgery), or with a poor communication with the outpatient, who can forget the scheduled date for the operation.^[37]^

Besides, inadequate preoperative preparation can occur when outpatients are not compliant with preoperative instructions regarding the fasting rules or their treatment management.^[38]^ A poor understanding of the instructions or memory impairment can be the cause of this non-compliance, which can harm patient safety during anesthesia care. Indeed, if the preoperative fasting rules are not respected, the patient risks suffering from a pulmonary aspiration.^[39]^

Moreover, even DOS cancellations rate are low in day surgery, they are still an issue for both healthcare structures and outpatients.^[35]^

Indeed, surgery cancellations can have significant emotional and economic effect for day surgery patients,^[40]^ which can lead to their dissatisfaction.^[38]^ Also, DOS cancellations can represent an important financial cost, by the waste of the human and material resources allocated to the planned surgery. Indeed, the average cost^[41]^ of one single cancelled operation varies widely across the world (from 30 US dollars in Brazil to 5000–8000 US dollars in USA).

This explains the needs for healthcare structures to reduce the DOS cancellations rate to the lowest. Thereby, in order to help outpatients retaining the preoperative instructions and following them, more and more hospitals and clinics use phone calls^[40]^ or SMS reminders the day before surgery.^[7,8,42]^ These interventions help to decrease DOS cancellations and meet outpatients’ satisfaction.^[43]^ However, DOS cancellations due to patients’ lack of compliance to preoperative instructions have decreased but are still happening.^[43,8]^

Day surgery implies breakthroughs in the care organization. In this context, preoperative information is the cornerstone, which enables:

1. healthcare providers to help patients coping with day surgery process and
2. outpatients to be active participant in their care through SDM and preoperative preparation.

Thereby, it is essential to meet outpatients’ needs to prevent DOS cancellations. However tailoring preoperative information is challenging in this context, because each outpatient differ from another.

To our knowledge, no study has investigated the patients’ capacities to be active participant through day surgery care, regarding their attributes (HL skills and preoperative anxiety levels) and their perception of the delivery of preoperative information.

## 3 Aims

This study is part of a research program, including 2 other studies. Its aim is to collect outpatients’ perception about the delivery of preoperative information and analyze their capacities to be involved in their care, regarding their preoperative anxiety and HL levels.

Therefore, this study will evaluate

1. different obstacles to outpatients’ engagement previously identified as physicians’ communication, HL and preoperative anxiety levels,
2. their effects on outpatients’ understanding of preoperative instructions.

This study also aims:

- To assess the real capacity of outpatient to be active participant through their day surgery pathway with their HL level evaluation;
- To investigate outpatients’ experience regarding preoperative information delivery through communication with physicians and SMS reminders;
- To investigate their level of preoperative anxiety;
- To assess outpatients’ understanding of preoperative instructions.

## 4 Methods and analysis

### 4.1 Design

This is a multicenter cross-sectional study, carried out in French public hospitals in Paris and its suburbs.

### 4.2 Participants/setting

#### 4.2.1 Selection criteria for the hospitals

**Figure 1:**
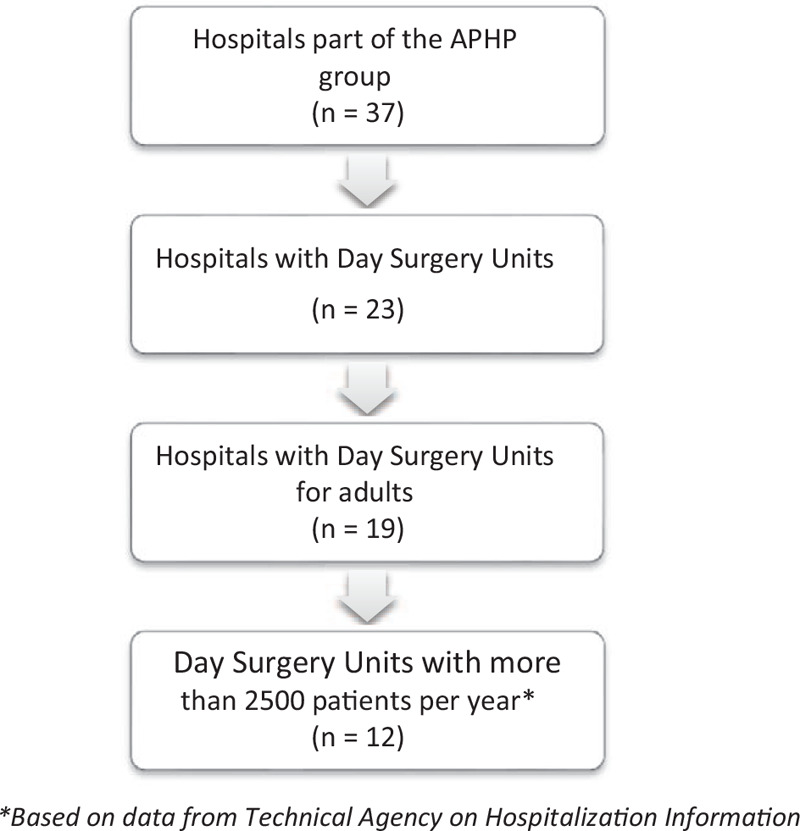
Selection criteria for eligible Day Surgery Units.

The study will take place in the hospitals of Assistance Publique-Hôpitaux de Paris (APHP), which use a SMS reminders system. Eligible institutions must meet the following inclusion criteria: having a day surgery unit (DSU), caring more than 2500 adult patients per year (Fig. 1). Considering these elements, 12 DSU will be included in this study. Therefore, in the eligible and selected DSU, managers will receive a letter inviting their healthcare unit to participate to this study. Phone calls will then be implemented in the recruitment process, and face-to-face visit in DSU for explaining the research project to the healthcare providers.

The choice of these institutions allows meeting patients with different social profiles and living in Paris and his suburbs. Recruitment will be carrying out from September 2021 to end to March 2022, covering 24 weeks.

#### 4.2.2 Patients’ inclusion and non-inclusion criteria

In this study, every outpatient in a DSU of an eligible APHP institution will be invited to participate to this study, following these criteria:

- Inclusion criteria: age >18 years old, scheduled day surgery (e.g., cataract surgery, upper and lower limb arthroscopy, appendectomy, varicose surgery…);
- Exclusion criteria: limited French proficiency, severe cognitive or hearing impairment, emergency surgery, upper and lower endoscopic procedures.

#### 4.2.3 Sample size

Because the representativeness is not a goal in this study, a non-probabilistic convenience sampling will be use, on the basis of 56,798 outpatients in the selected DSU in APHP structures for having a planned surgery in 2017.^[3]^

Within the framework of this study, it is planned to interview all the persons welcomed in the DSU in the 12 selected hospitals, about their experience after having received preoperative instruction, especially by SMS reminders, during 12 weeks in each institution. This total duration will correspond to 24 weeks. Its purpose is to provide evidence about outpatients’ perception of preoperative information delivery.

Thus, during the 24 weeks of inclusion time, 11688 patients are expected for outpatient surgery. In the hypothesis of estimated response rate of 60% in the presence of a facilitator,^[44]^ the number of patients to be included for this study is 7012 patients.

#### 4.2.4 Recruitment

The day of the scheduled surgery, in DSU, healthcare provider will verbally informed eligible patients about the study and will give them written information.

Then, in preoperative lounge, a healthcare provider or a member of the research team will propose to outpatients to participate in the study, if they agree.

#### 4.2.5 Data collection procedure

Participant will be invited to answer to an anonymous self-report questionnaire. Their experience will be assessed through Near Real Time Feedback, which consists of using new technologies to collect, analyze and evaluate patient experience recently after their care.^[44]^ Therefore, the questionnaire will be available online on touch screens; through an e-CRF.

Data collection will occur the day of the surgery. The questionnaire will be submitted to outpatients during their waiting before entering operating room, in the preoperative room. Hospital staff and research team members will encourage and help outpatients to fill in the form. Advertisement materials will be set in waiting rooms of the DSU and of the preoperative lounge to encourage patient participation. The questionnaire items will be presented in French only. Only fully completed questionnaires will be considered for the study.

The questionnaire will be peer-reviewed, before being pre-tested by 5 patients in September 2020. These pre-tests will help to assess acceptability of the questionnaire by measuring outpatients’ responses rate and their experience completing the digital survey and the completion time of the questionnaire.

#### 4.2.6 Measures

To understand outpatients’ experience regarding the preoperative instructions’ delivery in day surgery, the closed-ended questions of this survey used will evaluate psychometric criteria, other than outpatients’ satisfaction like:

- Their HL level;
- Their preoperative anxiety level;
- Their experience with the physician communication and the way they perceive it;
- Their understanding of preoperative instructions and their perception of SMS reminders.

#### 4.2.7 Outpatients’ experience with doctor communication

Outpatients’ experience of psychological preoperative preparation will be assessed with a relational Patient Reported Experience Measures (PREMS), a self-report questionnaire. This e-CRF will allow outpatients to report on their experience with regard to preoperative information received from surgeons and anesthetists. The selected tool is the Doctor Patient Communication scale,^[45]^ validated in French and used in acute conditions like emergency or surgery. Exploring the way physicians communicate with outpatients is essential in this study, because doctors’ low communication skills can contribute to preoperative anxiety.^[46]^

This PREMS includes 13 items, each is scored on a 4-point Likert scale from 1 (strongly disagree) à 4 (strongly agree). A score of 40 or above is considered as convenient.^[47]^

#### 4.2.8 Health literacy level

As argued, in day surgery, outpatients are expected to have different skills as being able to: engage in their healthcare, understand the information provided by staff, and communicate with them in order to take well-informed decisions regarding their care process. These complex abilities are contained in the concept of HL. Different tools can measure these skills, like the European Health Literacy Survey Questionnaire (HLS-EU-Q), a measure developed by a European Consortium.^[48]^ This self-report tool is based on the taxonomy of HL defined by SORENSEN.^[48]^ Through 47 items, it explores the 4 main dimensions of HL (accessing, understanding, appraising and applying healthcare information) and their application in 3 domains (healthcare, disease prevention and health promotion).^[48]^ A shorter version, the HLS-EU-Q16, including 16 items of the original 47 items from the HLS-EU-Q, has been developed to facilitate HL screening, especially in clinical settings.^[49]^

With a high correlation (*r* = . 82) with the 47 items of the HLS-EU-Q results, the HLS-EU-Q16 has been validated in French in 2018 by ROUQUETTE.^[50]^

In this study, this tool will enable to quickly identify outpatients’ health literacy skills. The scoring relies on the dichotomization of answers categories:

- “Fairly difficult” and “Very difficult” are both coded 0;
- “Fairly easy” and “Very easy” are coded 1;
- “Do not know” answers are coded as missing values

The HLS-EU-Q16 score is the sum score of each item, with a range from 0 to 16. Three levels of HL are defined according to this score: inadequate HL (0-8), problematic HL (9–12) and sufficient HL (13–16).

#### 4.2.9 Preoperative anxiety

It will be evaluated with the Amsterdam Preoperative Anxiety and Information Scale (APAIS), which is a self-report questionnaire including 6 questions.^[51]^

The scale explores three different domains of preoperative anxiety: anesthesia-related anxiety and surgery-related anxiety (for items 1, 2, 4, and 5) and need for information (items 3 and 6). The sum of the items related to anxiety defines a global anxiety score.

Each item is scored on a 5-point Likert scale and can be answered using the following options:

1. strongly disagree;
2. disagree;
3. quite disagree;
4. quite agree, and;
5. strongly agree.

Patients with a global anxiety score of 11 or above are considered as anxious.

The APAIS has already been used in several international studies in clinical area. A French version of the APAIS has been published and validated.^[51]^

#### 4.2.10 Outpatients’ understanding of preoperative instructions and perception of SMS reminders

In this study, it is important to assess how well outpatients understand preoperative instructions delivered by physicians and SMS reminders before surgery. Patients with low health literacy skills are less compliant for following preoperative instructions,^[13]^ which can lead to delays or cancellations of the surgery, especially, in day surgery context, outpatients are fully responsible of their preoperative care.

Therefore, outpatients’ understanding of preoperative instructions and their perception of SMS reminders will be assessed with closed-ended questions.

#### 4.2.11 Sociodemographic and clinical characteristics

Sociodemographic data including age, gender and education level will be collected. Clinical characteristics will be considered with the type of surgery act and previous day surgery care.

#### 4.2.12 Statistical analysis

Statistical analysis will be performed using an alpha level of 5% will be considered as significant with R software for Windows V.3.5.2. F. Prior to the main analysis, data normality will be assessed.

To sum up outpatients’ sociodemographic and clinical characteristics, descriptive statistics including percentages or means +/- Standard Deviation (SD) will be presented.

The distribution of total and subscale scores for health literacy, Doctor Patient Communication scale and APAIS will be described using means and medians with SDs and Interquartile Ranges (IQRs).

To assess outpatients’ health literacy skills, included participants sociodemographic data of will be compared according to their health literacy level. Thereby, we will define different categories of outpatients’ profile regarding their health literacy level.

Then, linear regressions will be performed and will include as dependent variables the outpatients’ perception of physicians’ communication, the preoperative anxiety levels, the outpatients’ understanding of preoperative instructions. These analyses will include participants’ relevant categories previously defined, regarding their sociodemographic and clinical characteristics and their health literacy levels.

## 5 Discussion

### 5.1 Strengths

Day surgery is nowadays a common practice worldwide, especially in France. Although it is widely recognized as a cost-effective and high-quality care,^[5]^ the shift represents by day surgery implies challenges for outpatients, healthcare providers and facilities. Indeed, the efficiency of this surgical pathway lies upon the association of three elements:

1. specific organization of healthcare institutions,
2. surgical act compatible with day surgery,
3. outpatients as active participants of their care.^[9]^

Actually, many national health agencies policies highly recommend patient involvement through SDM: in the USA with the Affordable Care Act,^[52]^ in the UK with National Institute for Health and Care Excellence in England (NICE) recommendations,^[53]^ in France with the law of March 4th, 2002.^[54]^

In day surgery, previous studies found that outpatients need to be involved throughout all day surgery process, especially in the preoperative phase: from the schedule of the surgery and the preoperative preparation^[55]^ to the SDM^[56]^. Therefore, in day surgery process, outpatients and their caregivers are fully in charge of preoperative and postoperative care, which can be perceived as a burden by them^[57]^ and thus be stressful.^[58]^

This explains the paramount importance of preoperative information to help outpatients coping with their care in day surgery.

However, different barriers to information delivery and, thus to patient involvement can occur. Preoperative anxiety is identified as one of them, which can result from a lack of information,^[15]^ especially a lack of content. Indeed, due to day surgery context, healthcare providers are less available to meet patients’ individual expectations of information.^[59,60]^ Another obstacle is due to the quality of preoperative information.

Actually, the lack of personalization of preoperative information can lead to exclude patients with LHL skills from understanding preoperative instructions,^[13]^ which is another challenge for healthcare providers.^[13,61]^ Thereby, LHL can disrupt patient involvement as in SDM^[52]^ and can potentially lead to failures like DOS cancellations, which can be harmful for both outpatients and healthcare settings.

Therefore, in this study, we will explore the effectiveness of preoperative communication interventions in day surgery process from outpatients’ perspective, according to these 2 barriers and preoperative instructions understanding.

The preoperative anxiety level will be assessed with a validated scale, the APAIS, which is known as particularly relevant in perioperative context. This tool will enable to compare the results of this study with the ones from other countries.

Regarding the evaluation of preoperative information quality, this study will explore outpatients’ experience with anesthetists and surgeons’ communication rather than their satisfaction. Indeed, satisfaction is not a reliable criterion^[5,62]^ for assessing patient experience, especially considering the way preoperative information is delivered by healthcare providers. We will use a relational PREMS, the Doctor Patient Communication scale,^[45]^ a validated measure in French and in English used in acute care.

Besides, with this research, preoperative information content will be explored considering outpatients’ capacities to understand it by evaluating patients’ understanding and functional literacy skills with the 16 items of the HLS-EU-Q16. This measure is important because, despite the growing use of SMS reminders^[43]^ and the preoperative information delivered by healthcare providers, lack of compliance with preoperative instructions decrease but still remains,^[8]^ which means SMS reminders are not fully effective.^[63]^ Moreover, this misunderstanding of preoperative rules can lead to like DOS cancellations, which can be harmful for both outpatients and healthcare facilities by threading patient safety.^[35,41]^ To assess the effectiveness of preoperative communication interventions (including SMS reminders), several questions, which are not part of a validated tool, will be added for exploring outpatients’ understanding of preoperative hygienic and fasting instructions.

Furthermore, the HLS-EU-Q16 will help us to gain insights about outpatients’ perception of their capability to get engaged in their health and in SDM process.

In this research, to increase the size of data collected in this quantitative study, different validated self-report questionnaires will be used to provide a evidence-based snapshot of outpatients’ population. Therefore, we will use Near Real Time Feedback (NRTF) to collect patients’ experience while they are still in hospitals.^[64]^

Digital tablets will be used to deliver questionnaires because it enables data to be assessed and analyzed faster than printed surveys.^[65]^ Indeed, the feedback about perioperative physicians’ communication is crucial because it will enable anesthetists and surgeons to improve the quality of preoperative information and SDM process, as “feedback is an informed, non-evaluative, objective appraisal of performance intended to improve clinical skills.”^[66]^ Therefore, feedback is helpful for healthcare providers but also for outpatients, because this study will help informing the gap in knowledge regarding outpatients’ capacities and may help healthcare providers in improving the design of preoperative information, especially by taking account of patients’ health literacy skills. Moreover, the use of digital surveys available on touch screens is a current practice in data collection about patients’ experience feedback.

### 5.2 Limitations

The questionnaire selected for collecting data for this study can be a point of discussion. Indeed, different response biases can occur with this tool, although different validated questionnaires will be used for this study.

Actually, despite the growing interest in Internet-based data collection in research, some patients rather use paper-based surveys, especially among older people aged 65 years and above,^[67]^ which can lead to a nonresponse bias and a lower expected response rate. Besides, some patients may show poor interests in providing feedback,^[67]^ unsure of the real impact of their answers on healthcare quality. Moreover, patients filling questionnaires sometimes care about giving answers making them “look good” ^[68]^ which is called the social-desirability bias. To prevent these different response biases, a member of the nurse team or of the research team will remind every participant the goal of the study and that every data collected will be strictly anonymous. Moreover, the facilitator will also provide additional explanations if needed regarding the use of the digital tablet and the wording of the questions, especially for outpatients with low health or digital literacy skills.

However, despite these different limitations, this study is the first, to our knowledge, exploring in depth outpatients’ experience regarding social (health literacy) and psychological factors (preoperative anxiety level).

## 6 Ethics and dissemination

This research adheres to the principles of the declaration of Helsinki.

Before proceeding to data collection, the managers of the selected hospitals should have given their consent. Following the oral consent and agreement, outpatient will be invited to answer to an anonymous self-report questionnaire. Before their participation, a letter will be given to participants, in order to inform them of the study's characteristics.

French ethics review committee (Comité d’Ethique de la Recherche) of the University of Paris has approved the study protocol (IRB 00012020-14). Besides, an authorization will be asked to the National Commission on Informatics and Liberty (Commission Nationale de l’Informatique et des Libertés) in order to guarantee data privacy. Indeed, every data collected will be anonymous, in order to respect the European policy with the General Data Protection Regulation.

Results from this study will be disseminated through oral communications and a scientific article in an international peer-reviewed journal.

## Author contributions

**Conceptualization:** Stephanie Chandler-Jeanville, Monique Rothan-Tondeur Monique.

**Methodology:** Stephanie Chandler-Jeanville, Ahouah Mathieu, Margat Aurore, Monique Rothan-Tondeur Monique.

**Writing – original draft:** Stephanie Chandler-Jeanville.

**Writing – review & editing:** Monique Rothan-Tondeur Monique.
